# Spillover effect of a dietary intervention on physical activity in a randomized controlled trial with colorectal cancer patients

**DOI:** 10.1186/s12966-025-01757-0

**Published:** 2025-05-09

**Authors:** Hege Berg Henriksen, Åshild Kolle, Andreas Stenling, Ingvild Paur, Siv Kjølsrud Bøhn, Pernille Brøto, Tuva Syrdal Tronstad, Rune Blomhoff, Sveinung Berntsen

**Affiliations:** 1 Department of Nutrition, Institute of Basic Medical Sciences, University of Oslo, Blindern, P.O. Box 1049, 0316 Oslo, Norway; 2 Division of Health Services, Norwegian Institute of Public Health, Oslo, Norway; 3 Department of Psychology, Umeå University, Umeå, Sweden; 4 Department of Sport Science and Physical Education, University of Agder, Kristiansand, Norway; 5 Norwegian Advisory Unit On Disease-Related Undernutrition, Oslo University Hospital, Oslo, Norway; 6 Department of Clinical Service, Division of Cancer Medicine, Oslo University Hospital, Oslo, Norway; 7 Biotechnology and Food Science, Norwegian University of Life Sciences, Ås, Norway; 8 Møre Og Romsdal Hospital Trust, Ålesund, Norway; 9 Department of Diet Care and Nutrition, Bærum Municipality, Bærum, Norway

**Keywords:** Spillover effect, Physical activity, Physical function, Dietary intervention, Colorectal cancer, Randomized controlled trial, Lifestyle, Behavior, Food-based dietary guidelines, National guidelines, Accelerometer

## Abstract

**Background:**

Randomized controlled studies (RCTs) targeting dietary changes may also lead to other, untargeted changes in lifestyle habits, as spillover effects. In particular, the isolated impact of the dietary intervention may be difficult to separate due to spillover effects from changes in physical activity and physical function. Therefore, the aim of this study was to investigate the spillover effect of a one-year dietary intervention in post-surgery colorectal cancer patients by comparing the changes in physical activity and physical function between the diet intervention group and the control group in a randomized controlled trial, called the CRC-NORDIET study.

**Methods:**

Men and women, aged 50–80 years were randomized into either the intervention group (*n* = 240) or the control group (*n* = 229). Both groups received similar incentives on physical activity. Activity sensors were used to collect data on physical activity at baseline, 6 months, and 12 months. Physical function was estimated by results from handgrip strength, 30 s sit-to-stand test and 6-min walking test. Anthropometric measurements and body composition were also measured.

**Results:**

We found a significantly higher increase in moderate-to-vigorous intensity physical activity (MVPA) of 0.18 h per day from baseline to 6 months in the diet intervention group compared to the control group, respectively. However, the spillover effect of the dietary intervention on physical activity diminished to 0.10 h per day at 12 months follow-up which was not statistically significantly different (*p* = 0.24) from the control group. All measures of physical function increased in both groups from baseline to 6 months with no further increase at the 12-month follow-up.

**Conclusions:**

The dietary intervention did not induce a significant spillover effect on physical activity after 12 months of baseline, which was the main timepoint of the intervention. Providing identical physical activity guidance to both study groups during the 12-month intensive dietary intervention period, ensured comparable levels of physical activity across both study groups. This approach facilitated the isolation and analysis of the dietary intervention's effects on primary endpoints, as well as effects of behaviour interventions in secondary preventions, such as the CRC-NORDIET study.

**Trial registration:**

The study is registered on the National Institutes of Health Clinical Trials website (www.ClinicalTrials.gov; Identifier: NCT01570010).

**Supplementary Information:**

The online version contains supplementary material available at 10.1186/s12966-025-01757-0.

## Introduction

Lifestyle risk behaviors, such as poor diet, physical inactivity, alcohol consumption and smoking, are associated with risk of developing non-communicable diseases (NCDs), like cancer, cardiovascular disease, and diabetes [1–4]. These associations are primarily based on results from observational studies, which are subjected to confounders and bias and therefore have limited strength of causality [5, 6]. Randomized controlled trials (RCT) are considered the golden standard for providing the highest strength of causal inference due to the equal distribution of confounding factors through randomization and the inclusion of a control group. However, for RCTs with lifestyle interventions, it is challenging to implement traditional controls, as used in pharmacological RCTs. This is mainly because finding an appropriate control for health behaviors is not straightforward [5, 6]. Therefore, instead of a traditional control group an alternative is to establish a control group that do not receive any intervention but instead continue with their habitual lifestyle. While such alternative control group may lack the rigor of a control, it is still a valuable approach to assess the impact of lifestyle interventions on behavior change and subsequent health outcomes [6].

When implementing a lifestyle intervention trial aiming at changing one type of risk behavior e.g. diet, it is possible that other risk behaviors can change simultaneously and having synergistic or spillover impacts on the risk of NDCs accordingly [7–10]. The spillover effect, in the context of Multiple-Health-Behavior-Change (MHBC) interventions, refers to the phenomenon where changes made in one health behavior influence changes in other health behaviors, often leading to broader improvements in overall health outcomes [7, 8]. Essentially, it suggests that when individuals successfully modify one behavior, such as quitting smoking or increasing physical activity, they may become more motivated or confident to address other health-related behaviors, such as improving their diet or managing stress. MHBC interventions have been used to reduce recourses for intervention implementation and health care costs and to increase motivation and self-efficacy to improve certain health risk behaviors. [7, 8, 11–13]. However, the effects of MHBC interventions are difficult to separate from spillover effects to untargeted behavior. For diet interventions in particular, changes in physical activity have been suggested to promote such spillover effects [7, 8, 13, 14].

While it is well-established that a healthy diet may reduce risk of several cancers, few studies have assessed the effect of a healthy diet on long term health outcomes after primary treatment. Thus, there is a need for high quality and well-designed intervention studies evaluating the effectiveness of diet behavior in cancer survivors [6, 10, 15, 16].

In the present study and based on the CRC-NORDIET study we investigated the spillover effect of a dietary intervention on changes in moderate-to-vigorous intensity physical activity (MVPA) (hours per day and steps per day) in colorectal cancer (CRC) patients after 6 and 12 months follow up. Moreover, we also investigated the spillover effect of the dietary intervention on physical function in CRC patients after 6 and 12 months follow up.

## Methods

### Subjects and study design

The Norwegian Dietary Guidelines and Colorectal Cancer Survival (CRC-NORDIET) study was an RCT aimed to investigate the effect of a healthy diet according to the national recommendations on disease free- and overall survival among colorectal cancer patients 5, 10 and 15 years after baseline [17]. The design was a multi-centre two-arms RCT, i.e. an intensive dietary intervention group and a control group receiving usual care. The intensive dietary intervention lasted for 12 months and with long-term following up of 14 additional years from baseline. Due to the fact of the high interrelationship between diet and physical activity, both the intervention group and the control group received similar general advice to promote physical activity.

The CRC-NORDIET study enrolled 503 participants aged 50 to 80 years, newly diagnosed with non-metastatic colorectal cancer staged (tumor-node-metastasis (TNM) I-III (ICD-10 codes C18-C20) between 2012–2020 at Oslo University Hospital and Akershus University Hospital. Eligibility required attendance at the first study visit within nine months of surgery, Norwegian literacy, and provision of written informed consent to participate in the study. Exclusion criteria included dementia, mental impairments affecting comprehension of the study’s intervention, or participating in conflicting studies [17].

### Physical activity in the CRC-NORDIET intervention

Enrolled participants were randomly assigned to either an intervention or a control group. The intervention group received a 12-month intensive, personalized dietary intervention, following guidelines as outlined by Henriksen et al. [17] (Supplementary Material 1), in addition to recommendations for general physical activity aligned with national standards [18]. The control group received the same advice on physical activity, and general information about the Norwegian food-based dietary guidelines (FBDG) [17, 18].

All participants were recommended to aim for at least 150 min (i.e. 2.5 h) MVPA per week. They received a booklet containing practical guidance for integrating physical activity into their daily lives. In addition, they were encouraged to take advantage of local resources, such as health training centers and swimming pools, to enhance their physical activity regimen [17].

In collaboration with"Active against cancer,"a nonprofit organization founded in 2007, the CRC-NORDIET study provided participants 6 months of complimentary access to the exercise facility “Pusterommet”. Located in several Norwegian hospitals, “Pusterommet” offers group training, yoga-classes, strength- and cardiovascular training. Additionally, physical therapists provide personalized exercise guidance, customizing activities to support each patient's rehabilitation process during and after cancer treatment. Participants were invited to attend an inspiration day within the first 12 months of the intervention. This event included a lecture on daily physical activity and interactions with physical therapists from “Pusterommet” [17].

### Assessment of physical activity

Data on physical activity were collected at baseline and after 6 and 12 months of follow-up. The Sensewear Mini Armband (BodyMedia, Pittsburgh, Pennsylvania, USA) recorded daily physical activity levels, sedentary time, and energy expenditure [19–21]. The activity monitor uses a combination of sensors to measure various physiological parameters, including heat flux, galvanic skin response, tri-axial acceleration, and skin temperature. Algorithms combine various activity intensities to estimate energy expenditure in metabolic equivalents (METs), defined to 3.5 ml O_2_ per kg body weight x minutes [22]. Physical activity was categorized as light (1.5–3 METs), moderate (3–6 METs), vigorous (> 6 METs), and moderate-to-vigorous intensity (> 3 METs). Sedentary time included all activities < 1.5 METs, including nighttime (midnight to 6:00 a.m.). Intensities are recorded in 1-min intervals, with the total duration measured in hours per day [23].

Participants were instructed to wear the activity monitor on their non-dominant arm continuously for seven days after each study visit. Each monitor was individually programmed with demographic and biometric information, including age, weight, height, sex and smoking status. They were advised to adhere to their normal daily routines, except removing the monitor during water-based activities or if any contraindications occurred. Adequate data capture was defined as a minimum of 80% wear time over a 24-h period and minimum 4 consecutive days.

The recorded data was processed using the Sensewear Professional Software Version 7.0 BodyMedia Inc. (Pittsburg, Pennsylvania, USA). This analysis was conducted on a secure laptop, which was not connected to the internet to ensure data protection. All data in the study was safely stored in a secure server called TSD (Service for Sensitive Data), designed for storing and post-processing sensitive data in compliance with the Norwegian “Personal Data Act” and “Health Research Act”. TSD is developed and maintained by IT-Department (USIT) at the University of Oslo, Norway. Participants with pacemakers were excluded from this measurement [17].

### Physical strength and function

Handgrip strength, a common indicator of overall body strength, was measured using MAP 80 K1 Handgrip dynamometer (KERN & SOHN GmbH, Balingen, Germany) according to the manufacturer’s protocol [17]. Women performed the tests using 40 kg springs, whereas men used 80 kg springs. Seated with elbows at a 90-degree angle, participants performed three maximum-effort grips with each hand, and the highest value was documented.

The 30-s sit-to-stand test (30STS) assessed lower body muscle endurance. A 44 cm tall chair, with no armrest, was used for all measurements. The participant started from a seated position, arms crossed at the chest and the legs parallel to the ground. The total number of full stands within 30 s was registered [17].

The 6-min’ walk test (6MWT) measured aerobic capacity and stamina. The test's purpose is to assess the distance an individual can walk within 6 min. The test was conducted in a straight, 30-m-long corridor with level surface. Pulse rate was monitored before and after the test, and subjective effort was quantified using the Borg Rating of Perceived Exertion scale [17].

### Anthropometric measurements

Height and weight were measured at each study visit using a digital measuring station known as Seca 285 (Seca Birmingham, United Kingdom) [24]. The measurements were carried out dressed in light clothing with no shoes, as elaborated in detail in Henriksen 2017 [17].

Waist circumference was measured at the midpoint between the top of the iliac crest and the lower margin of the last palpable rib [17]. Hip circumference was measured around the widest portion of the hips, with the subjects'feet positioned 12–15 cm apart [17].

### Body composition

Body composition assessment was conducted using the Lunar Dual-energy X-ray Absorptiometry (iDXA) machine (GE Healthcare Lunar, Buckinghamshire, United Kingdom) and the enCORE software version 18. High precision and valid measurements of body composition has been shown for this machine in a sub-population of the CRC-NORDIET study as well as in healthy subjects [25, 26]. Participants underwent the scanning process in a fasted state and dressed in light clothing. Certified and trained operators adhering to the protocols established by the International Society for Clinical Densitometry (ISCD) [27] performed all DXA scans. The scans provided measures for whole body fat mass, fat free mass and bone mineral density, which were included in the analysis.

### Descriptive and clinical data

Descriptive and clinical data, including age, sex, weight, height, TNM-status, tumor location, time and type of surgery and additional treatment, were obtained from the CRC-NORDIET database. Demographic and comorbidity questionnaires were used to describe marital status, education level, work ability and concurrent diseases [17].

### Statistical analysis

Linear mixed effects models with random intercepts were used to analyze the data. The linear mixed effects models were used to account for the nested data structure (i.e., time nested in individuals) and examine differences between the intervention and control group at the 6- and 12-months follow-up. Separate models were estimated for each of the primary (MVPA, steps) and secondary outcome variables (hand-grip strength, 6MWT, 30STS). An interaction term between treatment group and measurement point was included to examine differences between the intervention and control group (i.e., the treatment effect) from baseline to 6 months and from baseline to 12 months. The baseline values were assumed to be equal between the groups and are thus reflected in the intercept of the model [28]. Both unadjusted and adjusted models were estimated. In the adjusted models we adjusted for sex, education, TNM-status, age, time since surgery, and comorbidity as control variables. We used Stata version 18.0, maximum likelihood estimation, and an unstructured covariance structure when estimating the linear mixed effects models (Stata code for the models are presented in the Supplementary Material 2, Table S1. Under the assumption of missing at random, maximum likelihood estimation accommodates incomplete outcomes by including all available data in the estimation [29]. Hence, no imputation of missing data was conducted prior to the analyses. No sample size estimation was conducted specifically for the current exploratory study because this is secondary data analyses of the CRC-NORDIET study [17]. However, based on the primary outcome variables (MVPA and step count) in the current study, we included participants with physical activity data (MVPA and step counts) on at least one measurement point (i.e. at either baseline, 6- and/or 12-months following up. The graphical display of results presented in Fig. 2 are based on estimated marginal means from the adjusted linear mixed effects models.

We also conducted two sensitivity analyses to examine the impact of missing data on the results. The first sensitivity analysis only included cases with complete physical activity data across all three time points (*n* = 317). In the second sensitivity analysis we used the factored regressions approach with a partially factored specification that accounts for missing data in both outcome variables and predictor variables [29, 30]. Hence, these analyses include all 469 participants regardless of amount of missing data on predictor or outcome variables. The sensitivity analyses using the factored regressions approach were conducted in the software Blimp version 3.2.1 [31], which relies on Bayesian estimation and does not rely on a joint distribution of the analysis variables. Instead, in the factored regression approach the multivariate distribution is factored into the product of multiple univariate distributions. The models were estimated using 60,000 Markov Chain Monte Carlo iterations and 30,000 burn-in iterations, and a low potential scale reduction factor [32] was used as an indication of convergence (i.e., < 1.05). The results from the sensitivity analyses are briefly described in the results and presented in full in the supplemental material (see Supplementary Material 2 Tables S5-S14).

## Results

### Recruitment and baseline characteristics

**Fig. 1:**
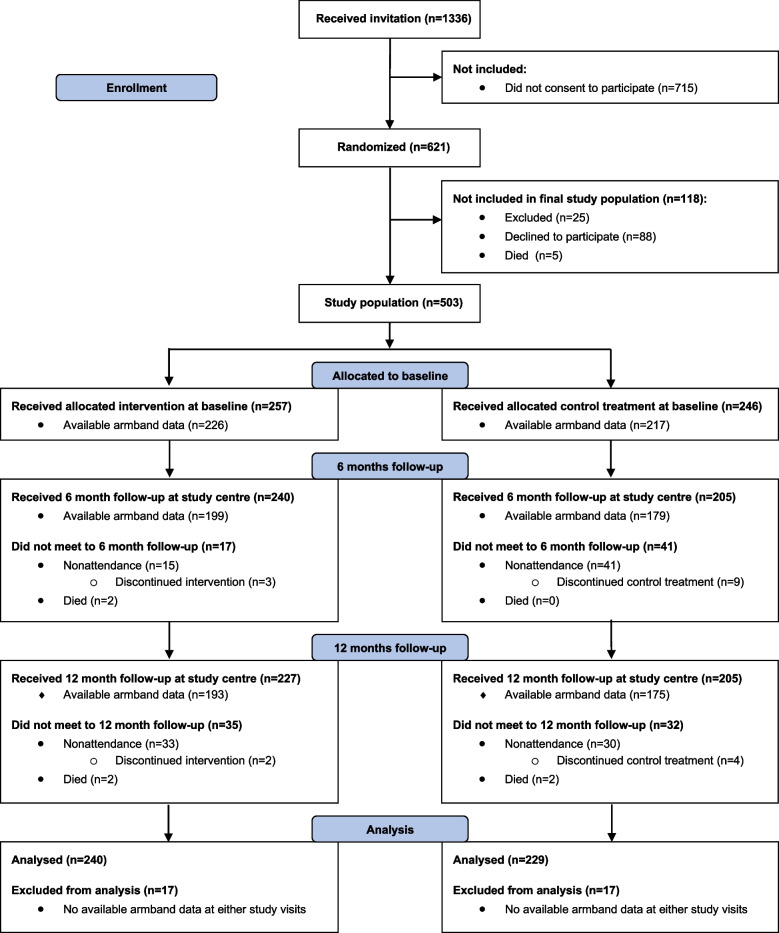
Flow of participants

The current study included 469 patients out of the original 503 enrolled in the CRC-NORDIET study and were used for the primary endpoint (Fig. 1). For two of the secondary outcome variables, i.e. the 6MWT and 30STS, the sample size was slightly lower due to additional missing data on these variables (394 and 455 participants, respectively).

Baseline characteristics are detailed in Table 1. In brief, of the included participants 250 (53.3%) and 219 (46.7%) were males and females, respectively. A mean age of 65 years and an average BMI of 27 kg/m^2^ were measured in both study groups. More than 60% of the subjects were classified as overweight or obese. The most frequent diagnosis was colon cancer (59%), with TNM stage II being predominant (~ 37%). More than 60% had at least one comorbidity, with musculoskeletal diseases being more prevalent in the intervention group (29%) than the control group (22%). Median MVPA was recorded as 509.8 and 468.0 min/week (or 8.5 and 7.8 h per week) in the diet intervention and control group, respectively. Average hand-grip strength, and 30STS were 33 kg and 15.6 stands in each study groups, respectively. The 6MWT did not differ between the study groups, with 584.4 and 576.1 m in the diet intervention av control group, respectively (Table 1).

**Table 1:** Baseline characteristics of the intervention and control group (*n* = 469)*

	**Intervention group (*n* = 240)**		**Control group (*n* = 229)**	
Male sex, n (%)	121	50.4	129	56.3
Age, years	65.2	7.3	65.9	7.9
Weight, kg	79.4	16.9	80.7	15.9
Height, cm	172.4	8.8	173.0	8.8
Waist circumference, cm	93.7	13.9	95.8	13.6
Hip circumference, cm	101.3	9.6	102.0	8.8
BMI, kg/m^2^	26.6	4.8	26.9	4.6
< *18.5*, n (%)	1	0.4	5	2.2
*18.5–24.9*, n (%)	87	36.3	72	31.4
*25.0–29.9*, n (%)	112	46.7	99	43.2
> *30.0*, n (%)	40	16.7	53	23.1
Tumor location, n (%)				
*C18 Colon*	141	59.0	133	58.6
*C19 Recto-sigmoid*	6	2.5	17	7.5
*C20 Rectum*	92	38.5	77	33.9
TNM stage^1^, n (%)				
*I*	77	32.1	71	31.0
*II*	93	38.8	82	35.8
*III*	70	29.2	76	33.2
Type of surgery, n (%)				
*Open*	66	27.7	69	30.3
*Laparoscopic*	145	60.9	129	56.6
*Laparoscopic converted to open*	17	7.1	19	8.3
*Endoscopic*	10	4.2	11	4.8
Additional treatment, n (%)				
*Neoadjuvant*	25	10.5	22	9.6
*Adjuvant*^***2***^	53	22.5	58	25.6
Ostomy, n (%)	63	26.3	58	25.4
Days between surgery and baseline	159.0	60.3	168.2	55.7
Comorbidity, n (%)				
*Any comorbidity*	161	67.1	148	64.6
*Musculoskeletal diseases*	70	29.2	52	22.7
*Heart diseases*	21	8.8	34	14.8
*Stroke*	11	4.7	3	1.3
*Diabetes*	24	10.0	28	12.2
*Other cancers*	58	24.7	52	22.8
*Other diseases*	53	22.1	74	32.3
Education, n (%)				
*Primary School*	23	9.7	22	9.7
*High School*	96	40.3	98	43.2
*University/College*	119	50.0	107	47.1
Working status, n (%)				
*Employed*	66	31.3	64	30.5
*Retired/unemployed*	145	68.7	146	69.5
Body composition				
*Fat mass, kg*	27.8	9.7	28.3	9.3
*Fat mass, %*	35.2	7.5	35.4	7.6
*Fat free mass, kg*	52.8	10.7	53.6	10.4
*Lean mass, kg*	50.0	10.1	50.9	9.9
Physical activity and tests				
*Handgrip strength, kg*	32.7	9.9	33.0	10.1
*30STS, stands*	15.6	4.9	15.6	4.9
*6MWT, meters*	584.4	94.2	576.1	98.6
*MVPA, minutes/week*, mean (min, max)	509.8	(278.8, 844.0)	468.0	(278.8,833.0)

### Primary outcomes – physical activity

**Fig. 2:**
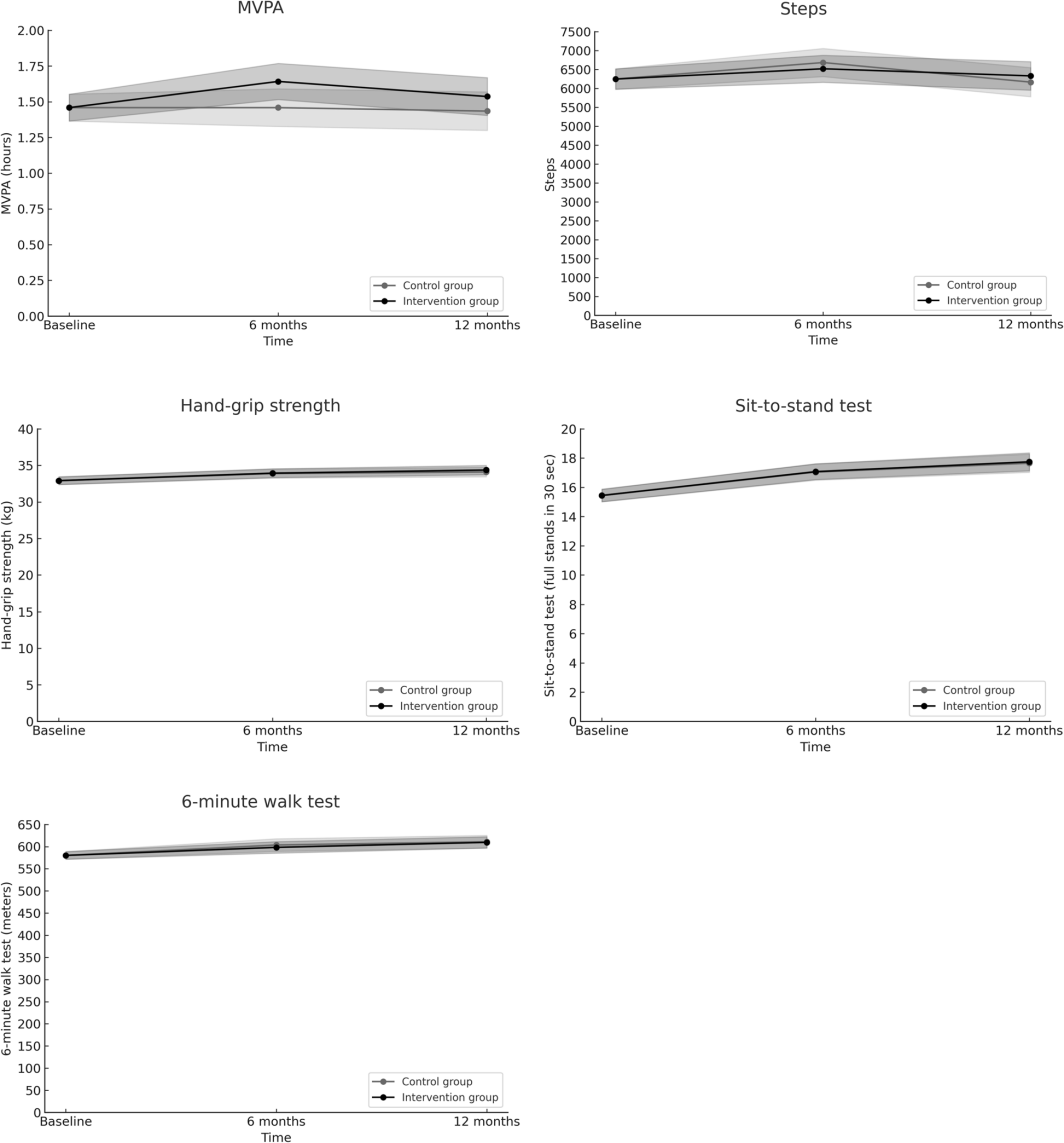
Differences in MVPA (h/d) (top left), steps (stands per day) (top right), hand-grip strength (kg) (mid left), 30STS (stands per 30 s) (mid right) and 6MWT (meters) (bottom left) across the three time points (baseline, 6 months, 12 months) between the diet intervention group (black dots) and the control group (grey dots)

At the 6 months follow-up, the diet intervention group had increased their MVPA compared to the control group as indicated by the statistically significant intervention group*measurement point (6 months) interaction effect (*γ* = 0.18, *SE* = 0.08, *p* = 0.03, 95% CI [0.02, 0.35]). However, at the 12-months follow-up this difference between the diet intervention and control groups in MVPA was reduced and not statistically significant (*γ* = 0.10, *SE* = 0.09, *p* = 0.24, 95% CI [−0.07, 0.27]) as indicated by the non-significant intervention group*measurement point (12 months) interaction effect. A graphical depiction of the differences in MVPA across the three time points is shown in Fig. 2 (top left) and the results are presented in Table 2.

**Table 2:** Unadjusted and Adjusted Linear Mixed Effects Model with MVPA as Dependent Variable

	Unadjusted (*n* = 469)					Adjusted (*n* = 464)				
	*γ*	*SE*	*p*	LL	UL	*γ*	*SE*	*p*	LL	UL
Intercept	1.45	0.05	0.00	1.35	1.55	1.57	0.10	0.00	1.38	1.76
Time										
6 months	0.00	0.06	0.96	−0.13	0.12	0.00	0.06	1.00	−0.12	0.12
12 months	−0.03	0.06	0.61	−0.16	0.09	−0.02	0.06	0.70	−0.15	0.10
Treatment*Time										
Intervention group*6 months	0.19	0.08	0.02	0.03	0.35	0.18	0.08	0.03	0.02	0.35
Intervention group*12 months	0.12	0.08	0.17	−0.05	0.28	0.10	0.09	0.24	−0.07	0.27
Control variables										
Sex (female)						−0.34	0.09	0.00	−0.51	−0.17
Education (university)						0.25	0.09	0.00	0.08	0.42
TNM 1 vs. TNM 2						−0.09	0.10	0.40	−0.29	0.12
TNM 1 vs. TNM 3						−0.16	0.11	0.15	−0.37	0.06
Age						−0.03	0.01	0.00	−0.04	−0.02
Time since surgery						0.00	0.00	0.01	0.00	0.00
Comorbidity						−0.04	0.08	0.62	−0.19	0.12

*Note*. *γ* = unstandardized regression coefficient, *SE* = standard error, *p* = *p* value, LL = 95% confidence interval lower limit, UL = 95% confidence interval upper limit

There were no statistically significant differences in steps between the diet intervention group and control group at the 6- (*γ* = −165.38, *SE* = 233.62, *p* = 0.48, 95% CI [−623.27, 292.50]) or 12-months (*γ* = 165.69, *SE* = 247.02, *p* = 0.50, 95% CI [−318.46, 649.84]) follow-up as indicated by the non-significant intervention group*measurement point interaction effects. A graphical depiction of the differences in steps across the three measurement points is shown in Fig. 2 (top right) and the results are presented in Table 3. Both groups increased the number of steps slightly from baseline to 6 months (average increase across both groups ≈ 354 steps), but at the 12 months follow-up the average number of steps across both groups were close to baseline values.

**Table 3:** Unadjusted and Adjusted Linear Mixed Effects Model with Steps as Dependent Variable

	Unadjusted (*n* = 469)					Adjusted (*n* = 464)				
				95% CI					95% CI	
	*γ*	*SE*	*p*	LL	UL	*γ*	*SE*	*p*	LL	UL
Intercept	6229.71	145.05	0.00	5945.42	6514.01	6203.34	277.23	0.00	5659.97	6746.71
Time										
6 months	393.50	174.76	0.02	50.98	736.01	437.04	176.50	0.01	91.10	782.98
12 months	−87.73	183.21	0.63	−446.81	271.36	−81.83	183.59	0.66	−441.66	278.00
Treatment*Time										
Intervention group*6 months	−92.51	232.66	0.69	−548.52	363.50	−165.38	233.62	0.48	−623.27	292.50
Intervention group*12 months	157.83	245.32	0.52	−322.99	638.65	165.69	247.02	0.50	−318.46	649.84
Control variables										
Sex (female)						−242.06	245.23	0.32	−722.71	238.59
Education (university)						764.22	245.23	0.00	283.58	1244.86
TNM 1 vs. TNM 2						−91.94	295.44	0.76	−670.98	487.11
TNM 1 vs. TNM 3						−551.51	309.41	0.08	−1157.94	54.91
Age						−129.92	16.15	0.00	−161.59	−98.26
Time since surgery						−2.32	2.15	0.28	−6.53	1.88
Comorbidity						172.49	223.48	0.44	−265.51	610.50

*Note*. *γ* = unstandardized regression coefficient, *SE* = standard error, *p* = *p* value, LL = 95% confidence interval lower limit, UL = 95% confidence interval upper limit

### Secondary outcomes – physical function

There were no statistically significant differences in hand-grip strength between the diet intervention group and control group at the 6- (*γ* = 0.06, *SE* = 0.30, *p* = 0.85, 95% CI [−0.53, 0.64]) or 12-month (*γ* = 0.27, *SE* = 0.34, *p* = 0.43, 95% CI [−0.40, 0.93]) follow-up as indicated by the non-significant intervention group*measurement point interaction effects. There was, however, a general increase in both groups from baseline to 12 months (average increase across both groups ≈ 1.3 kg) in hand-grip strength (see Table S2 in Supplementary Material 2 and Fig. 2 (mid left)).

There were no statistically significant differences in the 30STS between the diet intervention group and control group at the 6- (*γ* = 0.03, *SE* = 0.31, *p* = 0.93, 95% CI [−0.58, 0.63]) or 12-month (*γ* = 0.09, *SE* = 0.37, *p* = 0.80, 95% CI [−0.62, 0.81]) follow-up as indicated by the non-significant intervention group*measurement point interaction effects. There was, however, a general increase in both groups from baseline to 12 months (average increase across both groups ≈ 2.3 stands) in the 30STS (see Table S3 in ﻿Supplementary Material 2 and Fig. 2 (mid right)).

There were no statistically significant differences in the 6MWT between the diet intervention group and control group at the 6- (*γ* = −5.24, *SE* = 8.66, *p* = 0.55, 95% CI [−22.21, 11.73]) or 12-month (*γ* = −1.52, *SE* = 8.38, *p* = 0.86, 95% CI [−17.94, 14.90]) follow-up as indicated by the non-significant intervention group*measurement point interaction effects. There was, however, a general increase in both groups from baseline to 12 months (average increase across both groups ≈ 30 m) in 6MWT (see Table S4 in ﻿Supplementary Material 2 and Fig. 2 (bottom left).

### Sensitivity analyses

Results from the two sensitivity analyses are presented in Additional files Tables S5-S14. The two sensitivity analyses generally supported the findings from the main analyses and the effects on MVPA, hand-grip strength, 30STS, and 6MWT were almost identical (with minor differences in magnitude). One difference between the main findings and sensitivity analyses were observed in the complete case analyses for the main effect on steps at 6 months, which was weaker and not statistically significant in the complete case analyses (*γ* = 253.94, *SE* = 194.69, *p* = 0.19). However, although the main effect of time on steps was not statistically significant, it was in the same direction as the main analysis but slightly weaker in magnitude.

## Discussion

In the current study, we investigated the possible spillover effect of a dietary intervention on physical activity in colorectal cancer patients 6- and 12 months after baseline. Moreover, we also investigated effect of the dietary intervention on physical function at the same time points. We found a higher significant increase in MVPA of 0.18 h per day from baseline to 6 months in the diet intervention group when compared to the control group. However, the effect of the dietary intervention on MVPA diminished after 12 months of baseline and was no longer significant different between the two study groups. All tests estimating physical function increased in both groups from baseline to 6 months. No further increase was observed at the 12-month follow-up. These results persisted after adjusting for sex, age, education, TNM-stages, time since surgery and comorbidities.

Controls for lifestyle interventions in RCTs have been found challenging, because a controlled lifestyle intervention may always have some effect on the participants behavior, like attention, changes in awareness of own health due to biological measurements and other monitoring of lifestyle habits [6]. It is therefore recommended to offer the controls either an alternative lifestyle intervention or usual care as in real-world practice, such as some lifestyle advice [6, 33]. Based on these findings, the CRC-NORDIET study provided comparable advice, which were presented in both written and oral formats, without implementing a specific exercise intervention. The guidance advised participants in both study groups to engage in physical activity, including strength training, at least twice a week, in accordance with the Norwegian national guidelines on physical activity. This study design enhanced our capacity to evaluate the effects of the dietary intervention on health outcomes while allowing for the monitoring of physical activity levels in both groups to control for potential confounding variables.

The CRC-NORDIET study was designed to explore the effect of a 12-months intensive dietary intervention on survival outcomes [17]. The result from the current study indicates that the design of the CRC-NORDIET study worked as intended, showed by no significant difference in physical activity between the study groups after 12 months of intervention. This is of great value for future studies estimating effects of the 12-months dietary intervention on both primary and secondary endpoints of dietary interventions like the CRC-NORDIET study. Moreover, these results may also be useful when implementing secondary prevention interventions among colorectal cancer survivors in clinical health care practices.

After completing cancer treatment, including surgery, chemotherapy, and radiation therapy, individuals often strive to regain their health and well-being as quickly as possible. Fast recovery after cancer treatment is of utmost importance. The significant higher increase in MVPA in the diet intervention group after 6 months, together with the observation that the control group has higher body weight, BMI, fat mass and visceral adipose tissue than compared to the intervention group [34] may suggest that they recover faster than the control group halfway through the diet intervention.

Spillover effects of lifestyle interventions have been shown reduced after long term follow up in other studies as well [33, 35, 36] and spillover effects to untargeted behaviors in Multiple behavior change interventions may occur and therefore important to investigate [7, 8]. In the Chapman`s review of lifestyle interventions, dietary changes can be achieved when the intervention is most intense, but will diminish afterwards in the long run [35].

Spillover effects on untargeted behaviors (e.g. diet) was also found in the CanChange study [36] investigating effects on a telephone-delivered health coaching 12-months intervention on physical activity and monitoring of dietary intakes. Gerstel et al. investigated the impact of a lifestyle intervention on body weight, metabolic syndrome parameters, nutrition and physical activity in home-care providers [33]. They found an intermediate effect of weight loss also among the controls after 6 months which was not maintained at 12 months of intervention. The I CAN study investigated the feasibility of a 12-months lifestyle intervention among cancer patients undergoing curative and palliative chemotherapy [37]. They found a significant increase in dietary habits and physical activity after 4 months of intervention, which reduced to baseline levels after 12 months follow-up [37]. This may be due to the “ceiling” effect also observed in other lifestyle interventions [38, 39] referring to a phenomenon whereby the potential for sustainable improvement in measured outcomes has reached its threshold for further improvements among the participants.

The CRC-NORDIET study included general advice for physical activity for both the diet intervention group and the control group, but no specific behavior intervention aiming at improvements in physical function. However, since physical function is an important health outcome among cancer patients, the CRC-NORDIET study monitored physical function by different tests. Therefore, the current study investigated possible spillover effects of the diet intervention on physical function, estimated by hand grip strength, 30STS and 6MWT, and found no differences between the study groups at neither time points. However, improvements in both groups were documented from baseline to 12-months follow-up.

### Strengths and limitations

The RCT design of the CRC-NORDIET study is unique within lifestyle interventions, as observational and mechanistic study design are dominated among cancer patients. The strength is in the randomization to an individualized dietary intervention or an active control group with similar advice on being physical active in both study arms. Moreover, since it takes time to change behavior and to develop health effects on disease and survival, the intensive dietary intervention had a long-term focus with a duration of 12 months, followed by a maintenance period of additional 14 years. This was based on experiences from other lifestyle interventions among cancer patients [35, 40–42]. By this design, effect of the dietary intervention on the survival outcomes are possible to be estimated. Objective measurement tools, like accelerometers, are increasingly used in lifestyle interventions and gives more accurate measures of activity than questionnaires [43–45]. The present study used accelerometers to measure physical activity, which gives more precise and accurate measurements on possible differences in physical activity between the study groups than by using self-reported data from questionnaires.

It is important to note that the activity sensors (SWA) used to measure physical activity across various intensity levels may lead to an overestimation of physical activity [21]. Participants wore the sensors continuously, both day and night, over a one-week period, facilitating the registration of all activities in one-minute intervals during this time frame. This methodology accounts for the higher duration of moderate- to- vigorous physical activity recorded compared to self-reported physical activity data obtained through questionnaires. National guidelines of physical activity are usually based on epidemiological studies using questionnaires as assessment tool for physical activity and to investigate the effect on health outcomes in the population.

A limitation in the current study is the dropouts on the visits after 6 and 12 months follow up, which were higher in the control group compared to the diet intervention group. By using the linear mixed model analysis, we included participants with physical activity data on at least one measurement point and the assumption of missing at random and accommodates incomplete outcomes by including all available data in the estimation. The participants in the study were higher educated compared to the general Norwegian population [46]. However, the distribution of colon and rectal cancer cases, as well as the TNM stages (I-III), within the CRC-NORDIET study population appears to reflect similar proportions observed in the general population of colorectal cancer patients in Norway in 2012 [47]. This is also the year when recruitment for the study commenced. Thus, the CRC-NORDIET study population may be representative (i.e. external valid) for all CRC patients with primary invasive CRC, TNM stages I-III and ages between 50–80 years old.

Based on the finding in this study, identifying the synergistic interplay of health risk behaviors, such as the spillover effect of a target behavior to an untargeted behavior, has great impact on the design of the study and the effect of intervention on health outcomes.

## Conclusions

In the current study, no spillover effect of the intensive dietary intervention on physical activity was found at 12 months follow-up, despite the significant effect at the 6 months follow-up (i.e. half-way of the dietary intervention). The number of steps increased in both study groups from baseline to 6 months, however, neither of these effects were maintained at the 12-month follow-up. Moreover, no statistically significant differences between the diet intervention and control group were observed in any of the physical function outcomes. Both groups showed an increase in all three physical function tests from baseline to 6 months and from baseline to 12 months, which indicates a general improvement in physical function across the intervention.

## Supplementary Information

Supplementary Material 1

Supplementary Material 2

## Data Availability

No datasets were generated or analysed during the current study.

## Abbreviations

MVPA: Moderate-to-vigorous physical activity
30STS: 30 Seconds sit-to-stand test
6MWT: 6 Minutes walking test
PG-SGA: Patient-Generated Subjective Global Assessment
TNM: Tumor Node Metastasis
BMI: Body Index Mass
RCT: Randomized controlled trials
NCD: Non-communicable diseases
CRC-NORDIET study: The Norwegian Dietary Guidelines and Colorectal Cancer Survival study
FBDG: Food-based dietary guidelines
METs: Energy expenditure in metabolic equivalents
DXA: Lunar Dual-energy X-ray Absorptiometry
ISCD: International Society for Clinical Densitometry
