# Supplementary material for: Spillover effect of a dietary intervention on physical activity in a randomized controlled trial with colorectal cancer patients
Source: Int J Behav Nutr Phys Act. 2025 May 9;22:54. doi: 10.1186/s12966-025-01757-0 (PMC12063259; doi:10.1186/s12966-025-01757-0)
Supplement: Supplementary file 1 — Supplementary Material 1 [file 12966_2025_1757_MOESM1_ESM.docx]

**Additional file 1. The CRC-NORDIET intervention program [17]**

|  | **Intervention group** | **Control group** |
| --- | --- | --- |
| Dietary counselling by a registered clinical dietitian | Individual counseling at all visits at the study center.  Telephone dietary counseling between visits during the first year of intervention.  Telephone dietary counseling once a year during the maintenance period of intervention | No dietary intervention, only general dietary advice as part of the standard care |
| Discount-card | A discount card with 25% discount on all fresh vegetables, fruit, berries and fish and several healthy foods available during the first year of intervention |  |
| Delivery of free foods items | Delivery of free healthy food items at every visit at study center during the first 12 months of intervention.  Delivery of a box with free food items to their homes two times during the first 12 months of intervention |  |
| Cooking course | A one-day cooking course arranged by registered dietitians following a protocol in accordance with the NFBDG |  |
| CRC-NORDIET webpage | A log-in restricted dynamic webpage containing extensive information regarding the NFBDG, dietary advice, recipes, week menus and portion sizes.  The webpage is available to the end of study and it is continuously updated with new recipes in accordance to the NFBDG |  |
| Inspiration days | The inspiration day contained:  - Lectures about the NORDIET study  - Diet: practical demonstrations of foods (fruits, vegetables, whole-grain products) and portion sizes according to the NFBDG performed by registered clinical dietitians  - Physical activity: Lecture and practical demonstration by the physical therapists of home-based exercises to incorporate in daily life | The inspiration day contained:  - Lectures about the NORDIET study  - Physical activity: Lecture and practical demonstration by the physical therapists of home-based exercises to incorporate in daily life |
| Physical exercise | All the participants were offered free access to exercise facilities at “Pusterommet” (http://pusterommene.no/) during the first year of intervention. They also got individual counseling by a physiotherapist | |

*NFBDG: Norwegian food-based dietary guidelines

17. Henriksen HB, Ræder H, Bøhn SK, Paur I, Kværner AS, Billington S, et al. The Norwegian dietary guidelines and colorectal cancer survival (CRC-NORDIET) study: a food-based multicentre randomized controlled trial. BMC Cancer. 2017;17(1):83. Epub 2017/02/01. doi: 10.1186/s12885-017-3072-4. PubMed PMID: 28137255; PubMed Central PMCID: PMCPMC5282711.
