# Supplementary material for: Spillover effect of a dietary intervention on physical activity in a randomized controlled trial with colorectal cancer patients
Source: Int J Behav Nutr Phys Act. 2025 May 9;22:54. doi: 10.1186/s12966-025-01757-0 (PMC12063259; doi:10.1186/s12966-025-01757-0)
Supplement: Supplementary file 2 — Supplementary Material 2 [file 12966_2025_1757_MOESM2_ESM.docx]

Table S1. Stata Code

| Model | Stata code |
| --- | --- |
| MVPA |  |
| Unadjusted | mixed MVPA i.t2 i.t3 trx1_t2 trx1_t3 \|\| id: time, var cov(unstructured) |
| Adjusted | mixed MVPA i.t2 i.t3 trx1_t2 trx1_t3 ib(0).sex ib(0).educ ib(0).tnm ageGMC time_opGMC comorbCWC \|\| id: time, var cov(unstructured) |
|  |  |
| Steps |  |
| Unadjusted | mixed steps i.t2 i.t3 trx1_t2 trx1_t3 \|\| id: time, var cov(unstructured) |
| Adjusted | mixed steps i.t2 i.t3 trx1_t2 trx1_t3 ib(0).sex ib(0).educ ib(0).tnm ageGMC time_opGMC comorbCWC \|\| id: time, var cov(unstructured) |
|  |  |
| Hand-grip strength |  |
| Unadjusted | mixed hgs_max i.t2 i.t3 trx1_t2 trx1_t3 \|\| id: time, var cov(unstructured) |
| Adjusted | mixed hgs_max i.t2 i.t3 trx1_t2 trx1_t3 ib(0).sex ib(0).educ ib(0).tnm ageGMC time_opGMC comorbCWC \|\| id: time, var cov(unstructured) |
|  |  |
| Sit-to-stand test |  |
| Unadjusted | mixed sts i.t2 i.t3 trx1_t2 trx1_t3 \|\| id: time, var cov(unstructured) |
| Adjusted | mixed sts i.t2 i.t3 trx1_t2 trx1_t3 ib(0).sex ib(0).educ ib(0).tnm ageGMC time_opGMC comorbCWC \|\| id: time, var cov(unstructured) |
|  |  |
| 6-minute walk test |  |
| Unadjusted | mixed mwt i.t2 i.t3 trx1_t2 trx1_t3 \|\| id: time, var cov(unstructured) |
| Adjusted | mixed mwt i.t2 i.t3 trx1_t2 trx1_t3 ib(0).sex ib(0).educ ib(0).tnm ageGMC time_opGMC comorbCWC \|\| id: time, var cov(unstructured) |

**SECONDARY OUTCOMES**

Table S2. Unadjusted and Adjusted Linear Mixed Effects Model with Hand-Grip Strength as Dependent Variable

|  | Unadjusted (*n* = 469) | | | | |  | Adjusted (*n* = 465) | | | | |
| --- | --- | --- | --- | --- | --- | --- | --- | --- | --- | --- | --- |
|  |  |  |  | 95% CI | |  |  |  |  | 95% CI | |
|  | *γ* | *SE* | *p* | LL | UL |  | *γ* | *SE* | *p* | LL | UL |
| Intercept | 32.80 | 0.46 | 0.00 | 31.89 | 33.70 |  | 41.32 | 0.58 | 0.00 | 40.19 | 42.46 |
| Time |  |  |  |  |  |  |  |  |  |  |  |
| 6 months | 0.97 | 0.23 | 0.00 | 0.52 | 1.41 |  | 0.97 | 0.22 | 0.00 | 0.54 | 1.40 |
| 12 months | 1.21 | 0.24 | 0.00 | 0.73 | 1.69 |  | 1.19 | 0.25 | 0.00 | 0.70 | 1.67 |
| Treatment*Time |  |  |  |  |  |  |  |  |  |  |  |
| Intervention group*6 months | 0.07 | 0.31 | 0.81 | -0.53 | 0.68 |  | 0.06 | 0.30 | 0.85 | -0.53 | 0.64 |
| Intervention group*12 months | 0.13 | 0.34 | 0.69 | -0.53 | 0.79 |  | 0.27 | 0.34 | 0.43 | -0.40 | 0.93 |
| Control variables |  |  |  |  |  |  |  |  |  |  |  |
| Sex (female) |  |  |  |  |  |  | -16.19 | 0.52 | 0.00 | -17.21 | -15.17 |
| Education (university) |  |  |  |  |  |  | -0.16 | 0.52 | 0.76 | -1.18 | 0.86 |
| TNM 1 vs. TNM 2 |  |  |  |  |  |  | -1.75 | 0.63 | 0.01 | -2.98 | -0.52 |
| TNM 1 vs. TNM 3 |  |  |  |  |  |  | -0.78 | 0.66 | 0.23 | -2.07 | 0.50 |
| Age |  |  |  |  |  |  | -0.26 | 0.03 | 0.00 | -0.33 | -0.20 |
| Time since surgery |  |  |  |  |  |  | -0.01 | 0.00 | 0.20 | -0.01 | 0.00 |
| Comorbidity |  |  |  |  |  |  | 0.17 | 0.27 | 0.53 | -0.36 | 0.71 |

*Note*. *γ* = unstandardized regression coefficient, *SE* = standard error, *p* = *p* value, LL = 95% confidence interval lower limit, UL = 95% confidence interval upper limit

Table S3. Unadjusted and Adjusted Linear Mixed Effects Model with Sit-to-Stand Test as Dependent Variable

|  | Unadjusted (*n* = 455) | | | | |  | Adjusted (*n* = 453) | | | | |
| --- | --- | --- | --- | --- | --- | --- | --- | --- | --- | --- | --- |
|  |  |  |  | 95% CI | |  |  |  |  | 95% CI | |
|  | *γ* | *SE* | *p* | LL | UL |  | *γ* | *SE* | *p* | LL | UL |
| Intercept | 15.44 | 0.24 | 0.00 | 14.98 | 15.90 |  | 15.73 | 0.47 | 0.00 | 14.81 | 16.65 |
| Time |  |  |  |  |  |  |  |  |  |  |  |
| 6 months | 1.61 | 0.23 | 0.00 | 1.16 | 2.06 |  | 1.60 | 0.23 | 0.00 | 1.16 | 2.04 |
| 12 months | 2.25 | 0.27 | 0.00 | 1.72 | 2.79 |  | 2.21 | 0.26 | 0.00 | 1.69 | 2.72 |
| Treatment*Time |  |  |  |  |  |  |  |  |  |  |  |
| Intervention group*6 months | 0.03 | 0.32 | 0.93 | -0.59 | 0.65 |  | 0.03 | 0.31 | 0.93 | -0.58 | 0.63 |
| Intervention group*12 months | 0.02 | 0.38 | 0.95 | -0.72 | 0.77 |  | 0.09 | 0.37 | 0.80 | -0.62 | 0.81 |
| Control variables |  |  |  |  |  |  |  |  |  |  |  |
| Sex (female) |  |  |  |  |  |  | -2.43 | 0.42 | 0.00 | -3.26 | -1.59 |
| Education (university) |  |  |  |  |  |  | 2.19 | 0.42 | 0.00 | 1.36 | 3.03 |
| TNM 1 vs. TNM 2 |  |  |  |  |  |  | -0.28 | 0.51 | 0.59 | -1.28 | 0.73 |
| TNM 1 vs. TNM 3 |  |  |  |  |  |  | -0.51 | 0.53 | 0.34 | -1.55 | 0.54 |
| Age |  |  |  |  |  |  | -0.18 | 0.03 | 0.00 | -0.23 | -0.12 |
| Time since surgery |  |  |  |  |  |  | 0.00 | 0.00 | 0.79 | -0.01 | 0.01 |
| Comorbidity |  |  |  |  |  |  | -0.27 | 0.28 | 0.33 | -0.82 | 0.27 |

*Note*. *γ* = unstandardized regression coefficient, *SE* = standard error, *p* = *p* value, LL = 95% confidence interval lower limit, UL = 95% confidence interval upper limit

Table S4. Unadjusted and Adjusted Linear Mixed Effects Model with 6-Min Walk Test as dependent Variable

|  | Unadjusted (*n* = 394) | | | | |  | Adjusted (*n* = 391) | | | | |
| --- | --- | --- | --- | --- | --- | --- | --- | --- | --- | --- | --- |
|  |  |  |  | 95% CI | |  |  |  |  | 95% CI | |
|  | *γ* | *SE* | *p* | LL | UL |  | *γ* | *SE* | *p* | LL | UL |
| Intercept | 578.74 | 4.95 | 0.00 | 569.05 | 588.44 |  | 582.32 | 9.26 | 0.00 | 564.16 | 600.48 |
| Time |  |  |  |  |  |  |  |  |  |  |  |
| 6 months | 22.01 | 6.59 | 0.00 | 9.09 | 34.94 |  | 23.43 | 6.53 | 0.00 | 10.63 | 36.23 |
| 12 months | 30.25 | 6.51 | 0.00 | 17.49 | 43.01 |  | 30.98 | 6.41 | 0.00 | 18.41 | 43.54 |
| Treatment*Time |  |  |  |  |  |  |  |  |  |  |  |
| Intervention group*6 months | -3.32 | 8.78 | 0.71 | -20.53 | 13.90 |  | -5.24 | 8.66 | 0.55 | -22.21 | 11.73 |
| Intervention group*12 months | -0.76 | 8.51 | 0.93 | -17.45 | 15.92 |  | -1.52 | 8.38 | 0.86 | -17.94 | 14.90 |
| Control variables |  |  |  |  |  |  |  |  |  |  |  |
| Sex (female) |  |  |  |  |  |  | -42.85 | 8.47 | 0.00 | -59.46 | -26.24 |
| Education (university) |  |  |  |  |  |  | 49.33 | 8.46 | 0.00 | 32.74 | 65.91 |
| TNM 1 vs. TNM 2 |  |  |  |  |  |  | -12.52 | 10.10 | 0.22 | -32.31 | 7.27 |
| TNM 1 vs. TNM 3 |  |  |  |  |  |  | -9.36 | 10.70 | 0.38 | -30.32 | 11.61 |
| Age |  |  |  |  |  |  | -4.48 | 0.55 | 0.00 | -5.56 | -3.39 |
| Time since surgery |  |  |  |  |  |  | -0.12 | 0.08 | 0.13 | -0.27 | 0.03 |
| Comorbidity |  |  |  |  |  |  | -0.15 | 7.33 | 0.98 | -14.52 | 14.22 |

*Note*. *γ* = unstandardized regression coefficient, *SE* = standard error, *p* = *p* value, LL = 95% confidence interval lower limit, UL = 95% confidence interval upper limit

**SENSITIVITY ANALYSES – COMPLETE CASE ANALYSIS**

Table S5. Unadjusted and Adjusted Linear Mixed Effects Model with MVPA as Dependent Variable

|  | Unadjusted (*n* = 317) | | | | |  | Adjusted (*n* = 316) | | | | |
| --- | --- | --- | --- | --- | --- | --- | --- | --- | --- | --- | --- |
|  | *γ* | *SE* | *p* | LL | UL |  | *γ* | *SE* | *p* | LL | UL |
| Intercept | 1.51 | 0.06 | 0.00 | 1.39 | 1.63 |  | 1.61 | 0.12 | 0.00 | 1.38 | 1.84 |
| Time |  |  |  |  |  |  |  |  |  |  |  |
| 6 months | -0.02 | 0.07 | 0.75 | -0.16 | 0.11 |  | -0.02 | 0.07 | 0.80 | -0.16 | 0.12 |
| 12 months | -0.06 | 0.07 | 0.43 | -0.19 | 0.08 |  | -0.04 | 0.07 | 0.54 | -0.18 | 0.10 |
| Treatment*Time |  |  |  |  |  |  |  |  |  |  |  |
| Intervention group*6 months | 0.25 | 0.09 | 0.01 | 0.07 | 0.43 |  | 0.25 | 0.09 | 0.01 | 0.06 | 0.43 |
| Intervention group*12 months | 0.15 | 0.09 | 0.12 | -0.04 | 0.33 |  | 0.13 | 0.10 | 0.17 | -0.06 | 0.32 |
| Control variables |  |  |  |  |  |  |  |  |  |  |  |
| Sex (female) |  |  |  |  |  |  | -0.29 | 0.11 | 0.01 | -0.50 | -0.08 |
| Education (university) |  |  |  |  |  |  | 0.23 | 0.11 | 0.03 | 0.02 | 0.43 |
| TNM 1 vs. TNM 2 |  |  |  |  |  |  | -0.06 | 0.12 | 0.62 | -0.31 | 0.18 |
| TNM 1 vs. TNM 3 |  |  |  |  |  |  | -0.16 | 0.14 | 0.23 | -0.43 | 0.10 |
| Age |  |  |  |  |  |  | -0.03 | 0.01 | 0.00 | -0.04 | -0.01 |
| Time since surgery |  |  |  |  |  |  | 0.00 | 0.00 | 0.02 | 0.00 | 0.00 |
| Comorbidity |  |  |  |  |  |  | -0.06 | 0.09 | 0.51 | -0.22 | 0.11 |

*Note*. *γ* = unstandardized regression coefficient, *SE* = standard error, *p* = *p* value, LL = 95% confidence interval lower limit, UL = 95% confidence interval upper limit

Table S6. Unadjusted and Adjusted Linear Mixed Effects Model with Steps as Dependent Variable

|  | Unadjusted (*n* = 317) | | | | |  | Adjusted (*n* = 316) | | | | |
| --- | --- | --- | --- | --- | --- | --- | --- | --- | --- | --- | --- |
|  |  |  |  | 95% CI | |  |  |  |  | 95% CI | |
|  | *γ* | *SE* | *p* | LL | UL |  | *γ* | *SE* | *p* | LL | UL |
| Intercept | 6452.55 | 174.12 | 0.00 | 6111.29 | 6793.82 |  | 6490.92 | 324.13 | 0.00 | 5855.65 | 7126.20 |
| Time |  |  |  |  |  |  |  |  |  |  |  |
| 6 months | 215.85 | 192.71 | 0.26 | -161.86 | 593.56 |  | 253.94 | 194.69 | 0.19 | -127.65 | 635.52 |
| 12 months | -237.82 | 197.12 | 0.23 | -624.18 | 148.53 |  | -215.47 | 197.72 | 0.28 | -602.99 | 172.06 |
| Treatment*Time |  |  |  |  |  |  |  |  |  |  |  |
| Intervention group*6 months | 137.22 | 252.98 | 0.59 | -358.62 | 633.06 |  | 74.30 | 254.06 | 0.77 | -423.65 | 572.26 |
| Intervention group*12 months | 399.39 | 259.23 | 0.12 | -108.70 | 907.47 |  | 404.55 | 260.42 | 0.12 | -105.87 | 914.96 |
| Control variables |  |  |  |  |  |  |  |  |  |  |  |
| Sex (female) |  |  |  |  |  |  | -360.37 | 292.63 | 0.22 | -933.91 | 213.17 |
| Education (university) |  |  |  |  |  |  | 660.21 | 291.95 | 0.02 | 87.99 | 1232.43 |
| TNM 1 vs. TNM 2 |  |  |  |  |  |  | -71.98 | 341.76 | 0.83 | -741.82 | 597.85 |
| TNM 1 vs. TNM 3 |  |  |  |  |  |  | -532.99 | 371.04 | 0.15 | -1260.21 | 194.23 |
| Age |  |  |  |  |  |  | -118.29 | 19.03 | 0.00 | -155.59 | -81.00 |
| Time since surgery |  |  |  |  |  |  | -4.69 | 2.66 | 0.08 | -9.91 | 0.52 |
| Comorbidity |  |  |  |  |  |  | 130.77 | 236.35 | 0.58 | -332.47 | 594.02 |

*Note*. *γ* = unstandardized regression coefficient, *SE* = standard error, *p* = *p* value, LL = 95% confidence interval lower limit, UL = 95% confidence interval upper limit

Table S7. Unadjusted and Adjusted Linear Mixed Effects Model with Hand-Grip Strength as Dependent Variable

|  | Unadjusted (*n* = 317) | | | | |  | Adjusted (*n* = 316) | | | | |
| --- | --- | --- | --- | --- | --- | --- | --- | --- | --- | --- | --- |
|  |  |  |  | 95% CI | |  |  |  |  | 95% CI | |
|  | *γ* | *SE* | *p* | LL | UL |  | *γ* | *SE* | *p* | LL | UL |
| Intercept | 33.28 | 0.56 | 0.00 | 32.19 | 34.38 |  | 41.87 | 0.65 | 0.00 | 40.59 | 43.14 |
| Time |  |  |  |  |  |  |  |  |  |  |  |
| 6 months | 0.73 | 0.27 | 0.01 | 0.21 | 1.25 |  | 0.76 | 0.25 | 0.00 | 0.26 | 1.26 |
| 12 months | 0.99 | 0.27 | 0.00 | 0.46 | 1.53 |  | 1.00 | 0.28 | 0.00 | 0.46 | 1.54 |
| Treatment*Time |  |  |  |  |  |  |  |  |  |  |  |
| Intervention group*6 months | 0.35 | 0.36 | 0.34 | -0.36 | 1.06 |  | 0.32 | 0.34 | 0.35 | -0.35 | 0.99 |
| Intervention group*12 months | 0.29 | 0.37 | 0.43 | -0.44 | 1.02 |  | 0.37 | 0.37 | 0.32 | -0.36 | 1.10 |
| Control variables |  |  |  |  |  |  |  |  |  |  |  |
| Sex (female) |  |  |  |  |  |  | -16.21 | 0.60 | 0.00 | -17.39 | -15.04 |
| Education (university) |  |  |  |  |  |  | -0.06 | 0.60 | 0.92 | -1.23 | 1.11 |
| TNM 1 vs. TNM 2 |  |  |  |  |  |  | -1.99 | 0.70 | 0.00 | -3.36 | -0.62 |
| TNM 1 vs. TNM 3 |  |  |  |  |  |  | -1.34 | 0.76 | 0.08 | -2.83 | 0.14 |
| Age |  |  |  |  |  |  | -0.29 | 0.04 | 0.00 | -0.36 | -0.21 |
| Time since surgery |  |  |  |  |  |  | -0.01 | 0.01 | 0.04 | -0.02 | 0.00 |
| Comorbidity |  |  |  |  |  |  | 0.27 | 0.31 | 0.39 | -0.34 | 0.87 |

*Note*. *γ* = unstandardized regression coefficient, *SE* = standard error, *p* = *p* value, LL = 95% confidence interval lower limit, UL = 95% confidence interval upper limit

Table S8. Unadjusted and Adjusted Linear Mixed Effects Model with Sit-to-Stand Test as Dependent Variable

|  | Unadjusted (*n* = 316) | | | | |  | Adjusted (*n* = 315) | | | | |
| --- | --- | --- | --- | --- | --- | --- | --- | --- | --- | --- | --- |
|  |  |  |  | 95% CI | |  |  |  |  | 95% CI | |
|  | *γ* | *SE* | *p* | LL | UL |  | *γ* | *SE* | *p* | LL | UL |
| Intercept | 15.70 | 0.29 | 0.00 | 15.13 | 16.26 |  | 16.03 | 0.55 | 0.00 | 14.95 | 17.11 |
| Time |  |  |  |  |  |  |  |  |  |  |  |
| 6 months | 1.65 | 0.27 | 0.00 | 1.12 | 2.17 |  | 1.71 | 0.26 | 0.00 | 1.20 | 2.22 |
| 12 months | 2.39 | 0.31 | 0.00 | 1.78 | 3.00 |  | 2.37 | 0.30 | 0.00 | 1.79 | 2.95 |
| Treatment*Time |  |  |  |  |  |  |  |  |  |  |  |
| Intervention group*6 months | 0.11 | 0.36 | 0.76 | -0.60 | 0.82 |  | 0.04 | 0.35 | 0.91 | -0.65 | 0.73 |
| Intervention group*12 months | -0.05 | 0.42 | 0.90 | -0.88 | 0.78 |  | 0.01 | 0.41 | 0.98 | -0.78 | 0.81 |
| Control variables |  |  |  |  |  |  |  |  |  |  |  |
| Sex (female) |  |  |  |  |  |  | -2.50 | 0.51 | 0.00 | -3.50 | -1.51 |
| Education (university) |  |  |  |  |  |  | 2.25 | 0.51 | 0.00 | 1.25 | 3.24 |
| TNM 1 vs. TNM 2 |  |  |  |  |  |  | 0.10 | 0.59 | 0.87 | -1.06 | 1.26 |
| TNM 1 vs. TNM 3 |  |  |  |  |  |  | -1.06 | 0.64 | 0.10 | -2.32 | 0.20 |
| Age |  |  |  |  |  |  | -0.21 | 0.03 | 0.00 | -0.27 | -0.14 |
| Time since surgery |  |  |  |  |  |  | 0.00 | 0.00 | 0.85 | -0.01 | 0.01 |
| Comorbidity |  |  |  |  |  |  | -0.23 | 0.32 | 0.47 | -0.84 | 0.39 |

*Note*. *γ* = unstandardized regression coefficient, *SE* = standard error, *p* = *p* value, LL = 95% confidence interval lower limit, UL = 95% confidence interval upper limit

Table S9. Unadjusted and Adjusted Linear Mixed Effects Model with 6-Minute Walk Test as Dependent Variable

|  | Unadjusted (*n* = 281) | | | | |  | Adjusted (*n* = 279) | | | | |
| --- | --- | --- | --- | --- | --- | --- | --- | --- | --- | --- | --- |
|  |  |  |  | 95% CI | |  |  |  |  | 95% CI | |
|  | *γ* | *SE* | *p* | LL | UL |  | *γ* | *SE* | *p* | LL | UL |
| Intercept | 583.50 | 5.67 | 0.00 | 572.39 | 594.60 |  | 591.47 | 10.21 | 0.00 | 571.46 | 611.48 |
| Time |  |  |  |  |  |  |  |  |  |  |  |
| 6 months | 21.91 | 7.38 | 0.00 | 7.43 | 36.38 |  | 22.55 | 7.27 | 0.00 | 8.29 | 36.80 |
| 12 months | 28.74 | 7.11 | 0.00 | 14.80 | 42.67 |  | 29.32 | 6.98 | 0.00 | 15.64 | 43.00 |
| Treatment*Time |  |  |  |  |  |  |  |  |  |  |  |
| Intervention group*6 months | -3.54 | 9.63 | 0.71 | -22.43 | 15.34 |  | -4.42 | 9.45 | 0.64 | -22.94 | 14.11 |
| Intervention group*12 months | 0.54 | 9.14 | 0.95 | -17.38 | 18.46 |  | 0.38 | 8.97 | 0.97 | -17.19 | 17.95 |
| Control variables |  |  |  |  |  |  |  |  |  |  |  |
| Sex (female) |  |  |  |  |  |  | -46.50 | 9.59 | 0.00 | -65.30 | -27.70 |
| Education (university) |  |  |  |  |  |  | 42.79 | 9.53 | 0.00 | 24.11 | 61.47 |
| TNM 1 vs. TNM 2 |  |  |  |  |  |  | -6.53 | 11.07 | 0.56 | -28.23 | 15.17 |
| TNM 1 vs. TNM 3 |  |  |  |  |  |  | -16.35 | 12.24 | 0.18 | -40.34 | 7.64 |
| Age |  |  |  |  |  |  | -4.79 | 0.62 | 0.00 | -6.00 | -3.57 |
| Time since surgery |  |  |  |  |  |  | -0.17 | 0.09 | 0.06 | -0.34 | 0.01 |
| Comorbidity |  |  |  |  |  |  | 2.62 | 7.66 | 0.73 | -12.41 | 17.64 |

*Note*. *γ* = unstandardized regression coefficient, *SE* = standard error, *p* = *p* value, LL = 95% confidence interval lower limit, UL = 95% confidence interval upper limit

**SENSITIVITY ANALYSES – FACTORED REGRESSIONS APPROACH**

Table S10. Unadjusted and Adjusted Linear Mixed Effects Model with MVPA as Dependent Variable

|  | Unadjusted (*n* = 469) | | | | |  | Adjusted (*n* = 469) | | |
| --- | --- | --- | --- | --- | --- | --- | --- | --- | --- |
|  | *γ* | *SD* | LL | UL |  | *γ* | *SD* | LL | UL |
| Intercept | 1.45 | 0.05 | 1.35 | 1.55 |  | 1.58 | 0.10 | 1.39 | 1.77 |
| Time |  |  |  |  |  |  |  |  |  |
| 6 months | 0.00 | 0.06 | -0.12 | 0.12 |  | 0.00 | 0.06 | -0.12 | 0.12 |
| 12 months | -0.03 | 0.06 | -0.15 | 0.09 |  | -0.03 | 0.06 | -0.15 | 0.10 |
| Treatment*Time |  |  |  |  |  |  |  |  |  |
| Intervention group*6 months | 0.19 | 0.08 | 0.03 | 0.35 |  | 0.18 | 0.08 | 0.02 | 0.34 |
| Intervention group*12 months | 0.12 | 0.08 | -0.05 | 0.28 |  | 0.10 | 0.08 | -0.07 | 0.26 |
| Control variables |  |  |  |  |  |  |  |  |  |
| Sex (female) |  |  |  |  |  | -0.34 | 0.09 | -0.51 | -0.17 |
| Education (university) |  |  |  |  |  | 0.24 | 0.09 | 0.07 | 0.41 |
| TNM 1 vs. TNM 2 |  |  |  |  |  | -0.11 | 0.10 | -0.31 | 0.09 |
| TNM 1 vs. TNM 3 |  |  |  |  |  | -0.15 | 0.11 | -0.36 | 0.06 |
| Age |  |  |  |  |  | -0.03 | 0.01 | -0.04 | -0.02 |
| Time since surgery |  |  |  |  |  | 0.00 | 0.00 | 0.00 | 0.00 |
| Comorbidity |  |  |  |  |  | -0.06 | 0.08 | -0.21 | 0.09 |

*Note*. *γ* = posterior median, *SD* = standard deviation, LL = 95% credibility interval lower limit, UL = 95% credibility interval upper limit

Table S11. Unadjusted and Adjusted Linear Mixed Effects Model with Steps as Dependent Variable

|  | Unadjusted (*n* = 469) | | | | |  | Adjusted (*n* = 469) | | |
| --- | --- | --- | --- | --- | --- | --- | --- | --- | --- |
|  | *γ* | *SD* | LL | UL |  | *γ* | *SD* | LL | UL |
| Intercept | 6228.42 | 148.20 | 5938.14 | 6519.51 |  | 6209.80 | 280.03 | 5659.17 | 6752.15 |
| Time |  |  |  |  |  |  |  |  |  |
| 6 months | 394.31 | 175.44 | 49.86 | 742.00 |  | 415.74 | 176.37 | 68.60 | 760.94 |
| 12 months | -91.89 | 178.77 | -441.64 | 258.23 |  | -80.45 | 178.18 | -427.70 | 268.95 |
| Treatment*Time |  |  |  |  |  |  |  |  |  |
| Intervention group*6 months | -90.29 | 234.72 | -550.02 | 367.16 |  | -146.74 | 232.93 | -603.72 | 308.12 |
| Intervention group*12 months | 166.24 | 238.02 | -300.35 | 631.94 |  | 121.68 | 235.40 | -339.71 | 584.32 |
| Control variables |  |  |  |  |  |  |  |  |  |
| Sex (female) |  |  |  |  |  | -264.65 | 247.66 | -748.85 | 221.72 |
| Education (university) |  |  |  |  |  | 751.71 | 249.69 | 264.79 | 1243.36 |
| TNM 1 vs. TNM 2 |  |  |  |  |  | -133.23 | 298.12 | -708.88 | 462.88 |
| TNM 1 vs. TNM 3 |  |  |  |  |  | -509.43 | 316.04 | -1131.61 | 111.02 |
| Age |  |  |  |  |  | -128.90 | 16.50 | -161.47 | -96.38 |
| Time since surgery |  |  |  |  |  | -2.43 | 2.21 | -6.75 | 1.99 |
| Comorbidity |  |  |  |  |  | 107.18 | 217.86 | -322.61 | 529.74 |

*Note*. *γ* = posterior median, *SD* = standard deviation, LL = 95% credibility interval lower limit, UL = 95% credibility interval upper limit

Table S12. Unadjusted and Adjusted Linear Mixed Effects Model with Hand-Grip Strength as Dependent Variable

|  | Unadjusted (*n* = 469) | | | | |  | Adjusted (*n* = 469) | | |
| --- | --- | --- | --- | --- | --- | --- | --- | --- | --- |
|  | *γ* | *SD* | LL | UL |  | *γ* | *SD* | LL | UL |
| Intercept | 32.80 | 0.47 | 31.85 | 33.68 |  | 41.44 | 0.59 | 40.26 | 42.57 |
| Time |  |  |  |  |  |  |  |  |  |
| 6 months | 0.97 | 0.23 | 0.51 | 1.42 |  | 0.91 | 0.23 | 0.46 | 1.36 |
| 12 months | 1.21 | 0.23 | 0.76 | 1.67 |  | 1.15 | 0.23 | 0.70 | 1.60 |
| Treatment*Time |  |  |  |  |  |  |  |  |  |
| Intervention group*6 months | 0.08 | 0.32 | -0.54 | 0.70 |  | 0.18 | 0.31 | -0.43 | 0.79 |
| Intervention group*12 months | 0.15 | 0.32 | -0.48 | 0.77 |  | 0.26 | 0.31 | -0.35 | 0.87 |
| Control variables |  |  |  |  |  |  |  |  |  |
| Sex (female) |  |  |  |  |  | -16.26 | 0.52 | -17.29 | -15.24 |
| Education (university) |  |  |  |  |  | -0.25 | 0.52 | -1.28 | 0.75 |
| TNM 1 vs. TNM 2 |  |  |  |  |  | -1.83 | 0.63 | -3.07 | -0.59 |
| TNM 1 vs. TNM 3 |  |  |  |  |  | -0.74 | 0.67 | -2.07 | 0.57 |
| Age |  |  |  |  |  | -0.26 | 0.03 | -0.33 | -0.19 |
| Time since surgery |  |  |  |  |  | -0.01 | 0.00 | -0.02 | 0.00 |
| Comorbidity |  |  |  |  |  | 0.06 | 0.28 | -0.49 | 0.61 |

*Note*. *γ* = posterior median, *SD* = standard deviation, LL = 95% credibility interval lower limit, UL = 95% credibility interval upper limit

Table S13. Unadjusted and Adjusted Linear Mixed Effects Model with Sit-to-Stand Test as Dependent Variable

|  | Unadjusted (*n* = 469) | | | | |  | Adjusted (*n* = 469) | | |
| --- | --- | --- | --- | --- | --- | --- | --- | --- | --- |
|  | *γ* | *SD* | LL | UL |  | *γ* | *SD* | LL | UL |
| Intercept | 15.41 | 0.26 | 14.89 | 15.92 |  | 15.65 | 0.50 | 14.68 | 16.64 |
| Time |  |  |  |  |  |  |  |  |  |
| 6 months | 1.64 | 0.24 | 1.16 | 2.11 |  | 1.64 | 0.24 | 1.16 | 2.11 |
| 12 months | 2.27 | 0.24 | 1.79 | 2.74 |  | 2.27 | 0.24 | 1.80 | 2.75 |
| Treatment*Time |  |  |  |  |  |  |  |  |  |
| Intervention group*6 months | 0.03 | 0.32 | -0.61 | 0.67 |  | 0.03 | 0.32 | -0.59 | 0.66 |
| Intervention group*12 months | 0.05 | 0.33 | -0.59 | 0.70 |  | 0.03 | 0.33 | -0.61 | 0.67 |
| Control variables |  |  |  |  |  |  |  |  |  |
| Sex (female) |  |  |  |  |  | -2.55 | 0.44 | -3.41 | -1.70 |
| Education (university) |  |  |  |  |  | 2.47 | 0.44 | 1.60 | 3.34 |
| TNM 1 vs. TNM 2 |  |  |  |  |  | -0.37 | 0.53 | -1.41 | 0.67 |
| TNM 1 vs. TNM 3 |  |  |  |  |  | -0.39 | 0.56 | -1.49 | 0.70 |
| Age |  |  |  |  |  | -0.19 | 0.03 | -0.25 | -0.14 |
| Time since surgery |  |  |  |  |  | 0.00 | 0.00 | -0.01 | 0.01 |
| Comorbidity |  |  |  |  |  | -0.31 | 0.30 | -0.90 | 0.27 |

*Note*. *γ* = posterior median, *SD* = standard deviation, LL = 95% credibility interval lower limit, UL = 95% credibility interval upper limit

Table S14. Unadjusted and Adjusted Linear Mixed Effects Model with 6-Minute Walk Test as Dependent Variable

|  | Unadjusted (*n* = 469) | | | | |  | Adjusted (*n* = 469) | | |
| --- | --- | --- | --- | --- | --- | --- | --- | --- | --- |
|  | *γ* | *SD* | LL | UL |  | *γ* | *SD* | LL | UL |
| Intercept | 578.65 | 5.12 | 568.68 | 588.77 |  | 582.81 | 9.62 | 563.89 | 601.70 |
| Time |  |  |  |  |  |  |  |  |  |
| 6 months | 22.06 | 6.66 | 8.87 | 35.06 |  | 22.47 | 6.58 | 9.64 | 35.45 |
| 12 months | 30.27 | 6.42 | 17.79 | 42.90 |  | 30.79 | 6.26 | 18.54 | 43.07 |
| Treatment*Time |  |  |  |  |  |  |  |  |  |
| Intervention group*6 months | -2.13 | 8.80 | -19.49 | 15.16 |  | -4.19 | 8.73 | -21.34 | 12.82 |
| Intervention group*12 months | 1.16 | 8.29 | -15.11 | 17.16 |  | -0.94 | 8.01 | -16.50 | 14.79 |
| Control variables |  |  |  |  |  |  |  |  |  |
| Sex (female) |  |  |  |  |  | -43.54 | 8.67 | -60.90 | -26.97 |
| Education (university) |  |  |  |  |  | 47.58 | 8.80 | 30.40 | 64.97 |
| TNM 1 vs. TNM 2 |  |  |  |  |  | -14.20 | 10.24 | -34.40 | 5.58 |
| TNM 1 vs. TNM 3 |  |  |  |  |  | -7.12 | 10.87 | -27.89 | 14.51 |
| Age |  |  |  |  |  | -4.41 | 0.57 | -5.51 | -3.30 |
| Time since surgery |  |  |  |  |  | -0.15 | 0.08 | -0.30 | 0.00 |
| Comorbidity |  |  |  |  |  | -3.93 | 7.39 | -18.42 | 10.64 |

*Note*. *γ* = posterior median, *SD* = standard deviation, LL = 95% credibility interval lower limit, UL = 95% credibility interval upper limit
